# PLK1 is a critical determinant of tumor cell sensitivity to CPT11 and its inhibition enhances the drug antitumor efficacy in squamous cell carcinoma models sensitive and resistant to camptothecins

**DOI:** 10.18632/oncotarget.3538

**Published:** 2015-03-12

**Authors:** Valentina Zuco, Michelandrea De Cesare, Nadia Zaffaroni, Cinzia Lanzi, Giuliana Cassinelli

**Affiliations:** ^1^ Molecular Pharmacology Unit, Department of Experimental Oncology and Molecular Medicine, Fondazione IRCCS Istituto Nazionale dei Tumori, Milan, Italy

**Keywords:** CPT11, PLK1, BI2536, squamous cell carcinoma, combination treatment

## Abstract

Intrinsic and acquired tumor drug resistance limits the therapeutic efficacy of camptothecins (CPTs). Downregulation of the mitotic kinase PLK1 was found associated with apoptosis induced by SN38 (CPT11 active metabolite). We investigated the role of PLK1 in the cell response to CPTs in squamous cell carcinoma (SCC) and pediatric sarcoma cell lines and explored the therapeutic potential of the combination of CPT11 and the PLK1 inhibitor BI2536 in CPT-sensitive and -resistant tumor models. Gain- and loss-of-function experiments established a direct role for PLK1 in counteracting SN38 antiproliferative and pro-apoptotic effects. The ability to activate an efficient G2/M cell cycle checkpoint allowing PLK1 ubiquitination and degradation was found associated with SN38-induced apoptosis in SCC cells. However, the synergistic interaction between SN38 and BI2536 enhanced apoptosis in cell lines both sensitive and resistant to SN38-induced apoptotic cell death. A well-tolerated CPT11/BI2536 cotreatment resulted in improved antitumor effect against SCC xenografts in mice compared to single agent treatments. The increased apoptosis induction was reflected in a high rate of complete responses and cures in mice harboring SCC, including tumors with intrinsic or acquired resistance to CPTs. PLK1 inhibition represents a promising strategy to improve the antitumor efficacy of CPT11-based regimens.

## INTRODUCTION

Camptothecin (CPT) derivatives are potent cytotoxic drugs that have shown clinical activity in a variety of human tumors. The two CPTs currently approved in cancer therapy, topotecan (TPT) and irinotecan (CPT11), are used for treatment of glioblastomas, sarcomas and cancers of the cervix [1, 2]. CPT11, which functions as a pro-drug requiring conversion to the active metabolite SN38 by plasma and cellular carboxylesterases, is also used in gastrointestinal malignancies [1, 2]. As with most chemotherapeutics, CPTs are usually administered in combination with other agents and the discovery of new rational combinatorial strategies continues to be a goal in the effort to optimize the treatment therapeutic efficacy [3]. In fact, CPTs share with other cytotoxic drugs dose-limiting toxicities, which may prevent the use of effective doses. Additional limitations to the clinical efficacy of CPTs are related to tumor intrinsic and acquired drug resistance, which represent the main cause of therapeutic failure [2, 4].

CPTs’ activity relies on a highly specific mechanism of action. These drugs target with high selectivity DNA topoisomerase I (Top1) and, by docking at the enzyme-DNA interface, induce the formation of stable Top1-DNA cleavable complexes thus preventing DNA strand re-ligation. Following the collision of cleavable complexes with the replication or transcription machinery, Top1-linked DNA single-strand breaks can be converted to double-strand breaks which are responsible for the drug cytotoxic activity [2, 3, 5]. Drug induced double-strand breaks also trigger a DNA damage response characterized by activation of serine-threonine kinases driving the ATM-CHK2 and ATR-CHK1 mediated checkpoint pathways and cell cycle arrest at the G1/S and G2/M cell cycle phase transitions. Depending on the extent of DNA lesions, activation of DNA damage signaling results in DNA repair or programmed cell death [2]. Combination strategies able to promote tumor cell death may result in clinical benefit. Indeed, combining DNA damaging drugs with modulators of cell cycle checkpoints is an emerging approach pursued to improve therapeutic index and clinical efficacy [6].

Polo-like kinase 1 (PLK1) belongs to a family of serine/threonine kinases (PLK1-4) involved in cell cycle regulation [7, 8, 9]. PLK1 controls several steps of the cell cycle and is essential for the G2/M transition and cell division. In addition, it is a crucial component of the DNA damage response pathway. Its inactivation mediated by the ATM/ATR signaling is needed for induction of the G2/M checkpoint, whereas its kinase activity is required for checkpoint termination and cell cycle reentry following DNA damage arrest [8, 10-12]. *PLK1* overexpression, reported in several human tumor types, has been correlated with bad prognosis. These features make it an attractive target for cancer therapy [13-18]. Indeed, depletion of *PLK1* gene expression results in inhibition of proliferation due to accumulation in the mitotic phase and apoptosis induction in tumor cell lines [7, 8]. Among several small molecule PLK1 inhibitors developed in preclinical studies, a few, including the dihypteridinones BI2536 and BI6727 (volasertib), have entered clinical evaluation [18-22].

In a previous study, we observed that an early and significant apoptosis induction by the CPT ST1968 was associated with a marked reduction of PLK1 levels in human squamous and ovarian cancer cell lines [23]. Here, we explored the role of PLK1 in the sensitivity of cell lines of different tumor types to SN38 and evaluated pharmacological inhibition of PLK1 in preclinical models as an approach to enhance CPT11 antitumor activity and overcome drug resistance.

## RESULTS

### Downmodulation of PLK1 is a consistent feature of the apoptotic cell response to SN38

**Figure 1:**
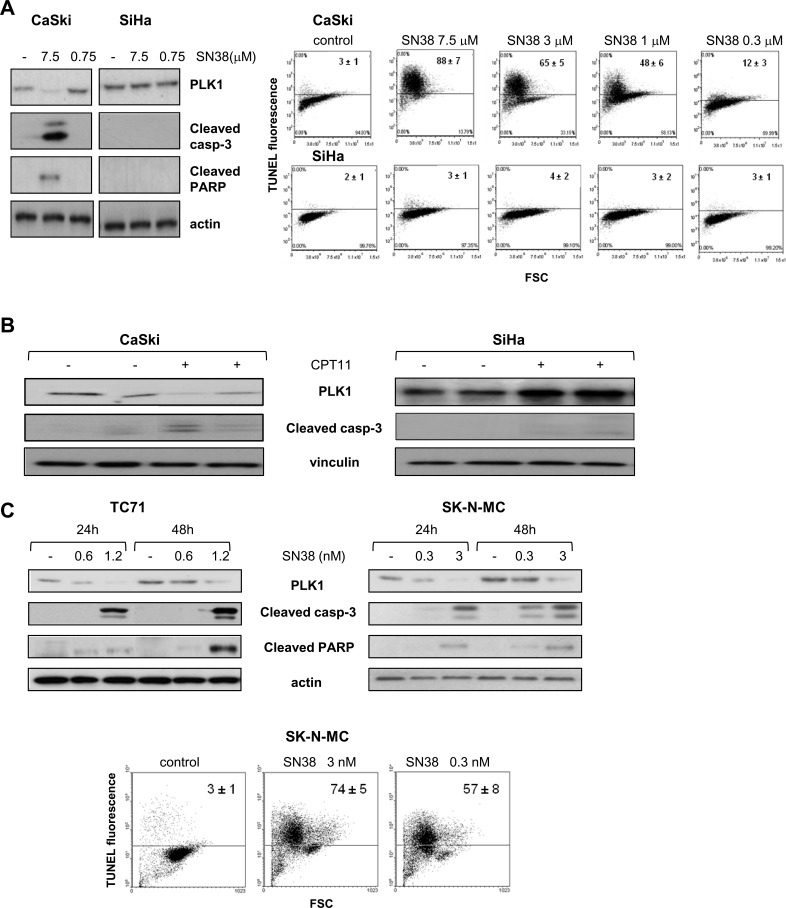
Modulation of PLK1 levels and apoptosis induction by SN38 A) The SCC cell lines CaSki and SiHa were exposed to the indicated concentrations of SN38 for 1h and analyzed by Western blotting (left panel), or TUNEL assay (right panel) after 24h or 72h, respectively. B) Mice bearing CaSki and SiHa tumors were treated with CPT11 (40 mg/kg i.p.). Twenty-four hours later, tumors were removed and processed for detection of PLK1 levels and cleaved caspase-3 by Western blotting. C) The ESFT cell lines TC71 and SK-N-MC were treated with SN38 concentrations corresponding to IC_50_ and IC_80_ values for each cell line. Upper panels, after 24 h and 48 h, cells were processed for Western blotting to analyze PLK1 levels and cleavage of caspase-3 and PARP. Lower panel, FACS analysis of TUNEL-positive SK-N-MC cells performed after 72h of exposure to SN38. Anti-vinculin or anti-actin blots show protein loading. In A) and C), one representative experiment is shown reporting mean percentages ± SD of TUNEL-positive cells from three independent experiments.

We investigated whether the relationship between drug-induced PLK1 downregulation and apoptotic cell death induction was a consistent event in tumor cell response to CPTs. To this aim, we examined the effect of treatment with SN38, the active metabolite of CPT11, in squamous cell carcinoma (SCC) cell lines previously characterized for sensitivity to the CPTs [24, 25]. Loss of PLK1 was observed after exposure to SN38 in CaSki cells, sensitive to CPT-induced apoptosis, and not in SiHa cells which are intrinsically resistant to SN38-induced apoptotic cell death as evidenced by Tunel assay performed on both SCC cell lines after treatment at equitoxic and equimolar concentrations (Suppl. Table 1 and Fig. 1A). Accordingly, downregulation of PLK1, associated with caspase-3 cleavage, was only found in lysates from CaSki tumor xenografts, grown sc in mice, after a single dose of CPT11 (Fig. 1B). These findings confirmed the relationship between PLK1 protein downregulation and apoptotic cell death in response to CPTs occurring both *in vitro* and *in vivo* in SCC models.

The association between the two events was further investigated in pediatric sarcoma cell lines as additional tumor models, since a role as survival kinase has been demonstrated for PLK1 in such tumor types [26, 27]. As shown in Fig. 1C, in the Ewing's sarcoma cells TC71 exposed to drug concentrations around the IC_50_ and IC_80_ [28] (and Suppl. Table 2), PLK1 downregulation paralled a remarkable apoptotic cell response evidenced by caspase-3 and PARP cleavage. Similar effects were observed in another Ewing's sarcoma family of tumors (ESFT) cell line, SK-N-MC. Apoptosis induction was further confirmed by a marked increase in the number of TUNEL–positive cells after SN38 treatment (Fig. 1C). Conversely, in the rhabdomyosarcoma cell line RD, less sensitive to the growth inhibitory activity of CPTs with respect to the ESFT cell lines [28] (and Suppl. Table 2), exposure to SN38 did not result in modulation of PLK1 protein levels or in apoptotic cell death (Suppl.Fig. 1A).

### SN38-induced PLK1 downregulation is a marker of efficient G2/M DNA damage checkpoint

**Figure 2:**
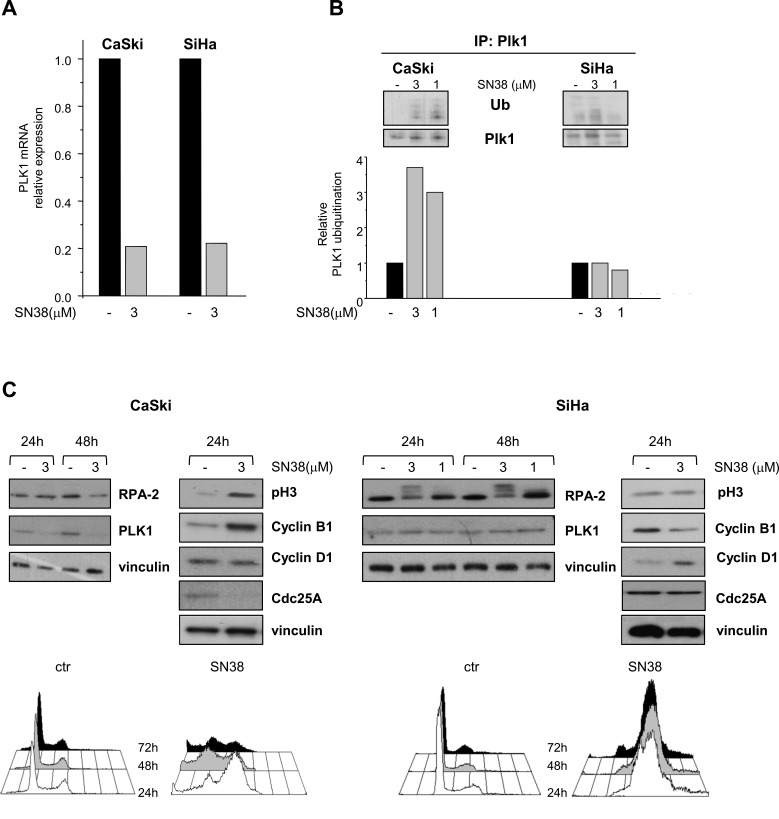
Effects of SN38 treatment on PLK1 transcription and ubiquitination and on cell cycle distribution in SCC cell lines CaSki and SiHa cells were treated for 1h with solvent (−) or equimolar concentrations of SN38. A) Twenty four hours later, cells were analyzed for PLK1 mRNA expression by qRT-PCR. B) In parallel, six hours after the end of treatment (1h), cell lysates were subjected to PLK1 immunoprecipitation and analyzed by Western blotting with anti-ubiquitin antibody (in upper panel representative blots are shown). Histograms represent the densitometric quantification of bands corresponding to ubiquitinated relative to not ubiquitinated PLK1 in each cell lines. Data from two independent experiments are expressed in arbitrary units referred to untreated cells (lower panel). C) Cells were exposed to solvent (−) or to the indicated concentrations of SN38 for 1h. Cell lysates were analyzed by Western blotting to evaluate the levels of RPA-2, PLK1, Cdc25A and cell cycle-specific markers. The hyperphosphorylated form of RPA-2 in SiHa cells was evidenced by the electrophoretic mobility shift. Vinculin is shown as a control of protein loading. Lower panels, cell cycle distribution of control and SN38 treated (3μM, for 1h) CaSki and SiHa cells, analyzed 24, 48 and 72 h after treatment.

Since both transcriptional and posttranslational mechanisms have been involved in the regulation of PLK1 expression [12, 29, 30], we investigated whether these regulatory processes could account for differences in PLK1 modulation in SCC cell lines. CPT-mediated transcriptional downmodulation of mitotic regulators, including PLK1, has been previously reported [31]. Quantitative RT-PCR results showed that PLK1 mRNA levels were decreased after 24h of SN38 treatment in both CaSki and SiHa cells (Fig. 2A). Immunoprecipitation of PLK1 from CaSki cells 6h after 1h of drug exposure evidenced a dose-dependent increase in the amount of ubiquitinated PLK1 (Fig. 2B). This finding was consistent with a functional G2/M DNA damage checkpoint promoting ubiquitination via cullin-based E3 ubiquitin ligases and subsequent proteasome-dependent degradation of crucial mitotic regulators such as the dual specificity phosphatase Cdc25A and PLK1 [11, 12, 30, 32]. Indeed, in CaSki cells, the downmodulation of PLK1 and Cdc25A was associated with increased levels of the mitosis markers phospho-Ser^10^ H3 and cyclin B1 (Fig. 2C). Analysis of cell cycle distribution evidenced in these cells a transient drug-induced accumulation of cells in G2/M phase preceding apoptosis induction (Fig. 2C and Suppl.Fig. 1B). In contrast, the lack of remarkable changes in the levels of PLK1 ubiquitination (Fig. 2B) and the absence of Cdc25A downmodulation observed in SiHa cells exposed to equimolar concentrations of SN38 (Fig. 2C), suggested a defective DNA damage checkpoint/signaling. Accordingly, the analysis of cell cycle distribution and of nuclear morphology evidenced a persistent drug-induced accumulation of SiHa cells with a 4N DNA content despite a low expression of cyclin B1 and phospho-Ser^10^ histone H3 (Fig. 2C and Suppl Fig. 1B). Furthermore, the hyperphosphorylation of RPA-2, associated with increased levels cyclin D1, indicated that SiHa cells had skipped the G2/M cell cycle phase, directly slipping into a G1-like phase despite the presence of persistent DNA damage. Similarly, in sarcoma cells, Cdc25A was downmodulated in the CPT-sensitive TC71 cells and not in RD cells in response to SN38 suggesting an impairment of DNA damage checkpoint in the resistant rhabdomyosarcoma cell line (Suppl. Fig. 1C). Moreover, Cdc25A appeared coherently modulated with PLK1 also in the *in vivo* setting in SCC from mice treated with CPT11 (Suppl. Fig. 1D). Thus, the failure to downregulate PLK1 through ubiquitilation and proteasome-dependent proteolysis appears to be associated with a less efficient CPT-induced G2/M checkpoint.

### Modulation of PLK1 expression affects cell sensitivity to SN38

**Figure 3:**
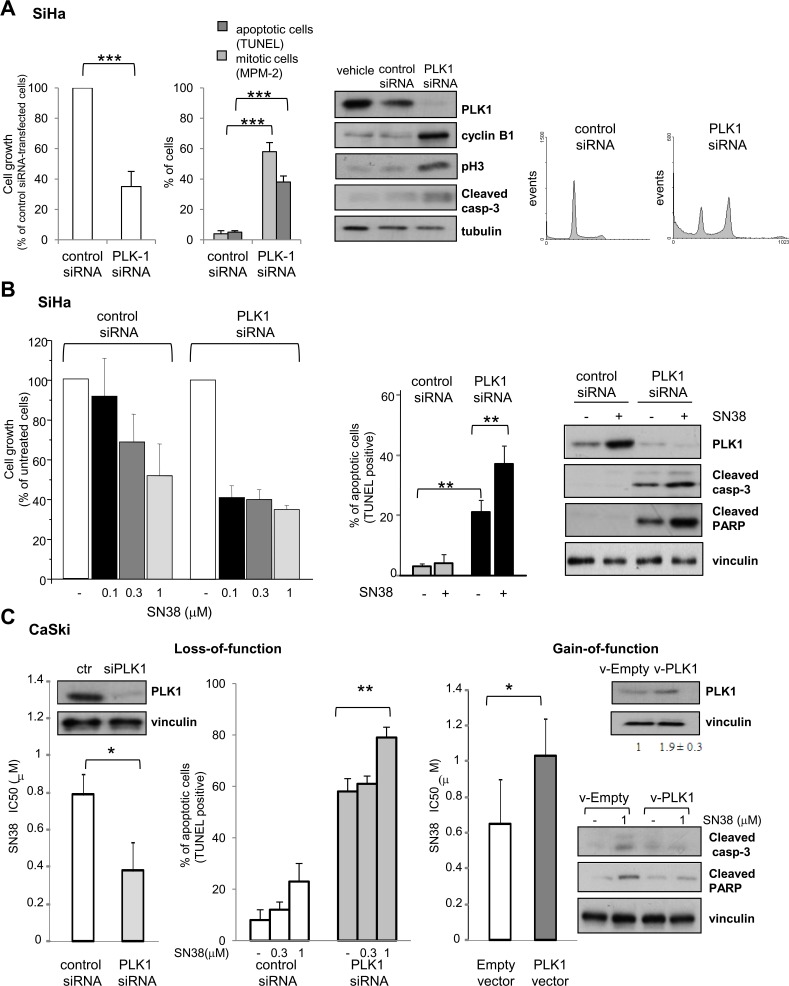
Effects of loss- and gain-of-function on SCC cell lines A) SiHa cells were treated with transfection reagent (vehicle), aspecific RNA oligonucleotide (control siRNA) or PLK1-directed siRNA (PLK1 siRNA). Left panel, the effect of PLK1 knockdown on cell growth (cell counting), induction of apoptosis (TUNEL assay) and mitotic cell number (MPM-2 detection by immunofluorescence) was assayed 72h after transfection. Values of cell growth are given in percentage ± SD referred to the negative control siRNA-transfected cells (100%). Central panel, cells were lysed 48h after transfection to assess levels of PLK1 and apoptotic or G2/M cell cycle phase specific markers by Western blot analysis. Tubulin is shown as a loading control. Right panel shows FACS analysis of DNA content and cell cycle distribution of cells stained with propidium-iodide 72h after transfection B) SN38 antiproliferative activity and apoptosis induction were examined in SiHa cells transiently transfected with control or PLK1-directed siRNA. Twenty four hours after transfection, cells were exposed to solvent (−) or to the indicated concentrations of SN38 for 1h. Three days after the end of treatment, the drug antiproliferative activity was evaluated by cell counting (left panel). Values are expressed as percentage ± SD of untreated cells (100%) from three independent experiments. Apoptosis was assessed by TUNEL assay (central panel) and Western blot analysis of PLK1 and cleavage of caspase-3 and PARP was performed in cells exposed to 3 μM SN38 (right panel). Protein loading is shown by vinculin. C) CaSki cells were transiently transfected with control or PLK1-directed siRNAs (Loss-of-function) or, alternatively, with empty or *PLK1*-expressing vector (Gain-of-function). Left, 24h after siRNA transfection, cells were exposed to SN38 for 72h to assess drug antiproliferative activity by cell counting. Apoptosis induction by SN38 was evaluated in siRNA-transfected cells by TUNEL assay 72h after treatment. Western blots show, on the left, levels of PLK1 after 72h of PLK1 siRNA transfection. Right, 24h after transfection with the *PLK1* expression vector, cells were exposed to SN38 and IC_50_s were calculated after 72h. Western blots in the upper panel show PLK1 levels after 72h of *PLK1* vector transfection. PLK1 bands were quantified using ImageJ software and normalized to vinculin. Values are expressed as arbitrary units referred to v-Empty-transfected cells (two independent experiments). In the lower panel, caspase-3 and PARP cleavage after 72h of SN38 treatment is shown (96h after transfection). Vinculin is shown as a control of protein loading. Columns and bars: mean percentage ± SD from three independent experiments. *P < 0.05; **P < 0.01, ***P<0.001 by Student's t test

To assess whether PLK1 directly contributes to the cellular outcome in response to SN38, we modulated *PLK1* expression in SCC cell lines. Fig. 3A shows that, in SiHa cells, PLK1 knockdown by siRNA resulted in a marked inhibition of cell growth (about 60%) and in the accumulation of mitotic and apoptotic cells. The occurrence of a mitotic arrest [33] was also supported by the enhancement of M phase markers (i.e. cyclin B1, phospho-Ser^10^ histone H3 and MPM-2) and by the accumulation of cells with 4N DNA content. The induction of apoptotic cell death by PLK1 silencing was confirmed by increased number of TUNEL positive cells and processing of caspase-3. Coherently, Hoechst nuclei staining showed the coexistence of aberrant mitoses and nuclei with apoptotic features in the silenced cell population (not shown). These data indicated that also in the CPT-resistant SiHa cells, PLK1 plays a prosurvival role and that reluctance of these cells to SN38 cytotoxicity was not related to defects in the apoptotic machinery.

We next investigated the impact of PLK1 downmodulation on SCC cell sensitivity to SN38. As shown in Fig. 3B, an increased CPT-induced antiproliferative activity was observed in PLK1 silenced SiHa cells, with a marked reduction of the IC_50_ (1μM in cells with control siRNA vs <0.1 μM in cells with PLK1 siRNA). This effect was associated with a significant enhacement of apoptosis detected by the TUNEL positivity and the proteolytic cleavage of caspase-3 and PARP in PLK1*-*silenced SN38-treated cells.

To get further insights into the role of *PLK1* expression levels in cell response to SN38, we applied a gain- and loss-of function approach to the CaSki cell line. As in SiHa cells, PLK1 dowmodulation by siRNA was able to enhance the antiproliferative and proapoptotic effects of SN38 (Fig. 3C). Coherently, a slight but reproducible increase of the mitotic kinase expression achieved by transient transfection with *PLK1* expression vector, determined a significant decrease of cell sensitivity to SN38, together with reduced caspase-3 and PARP cleavage, indicating a weakened apoptosis induction. These findings confirmed that PLK1 plays a direct role in determining the cellular outcome in response to CPT treatment.

### Pharmacological targeting of PLK1 kinase counteracts intrinsic and acquired resistance to SN38 *in vitro*

**Figure 4:**
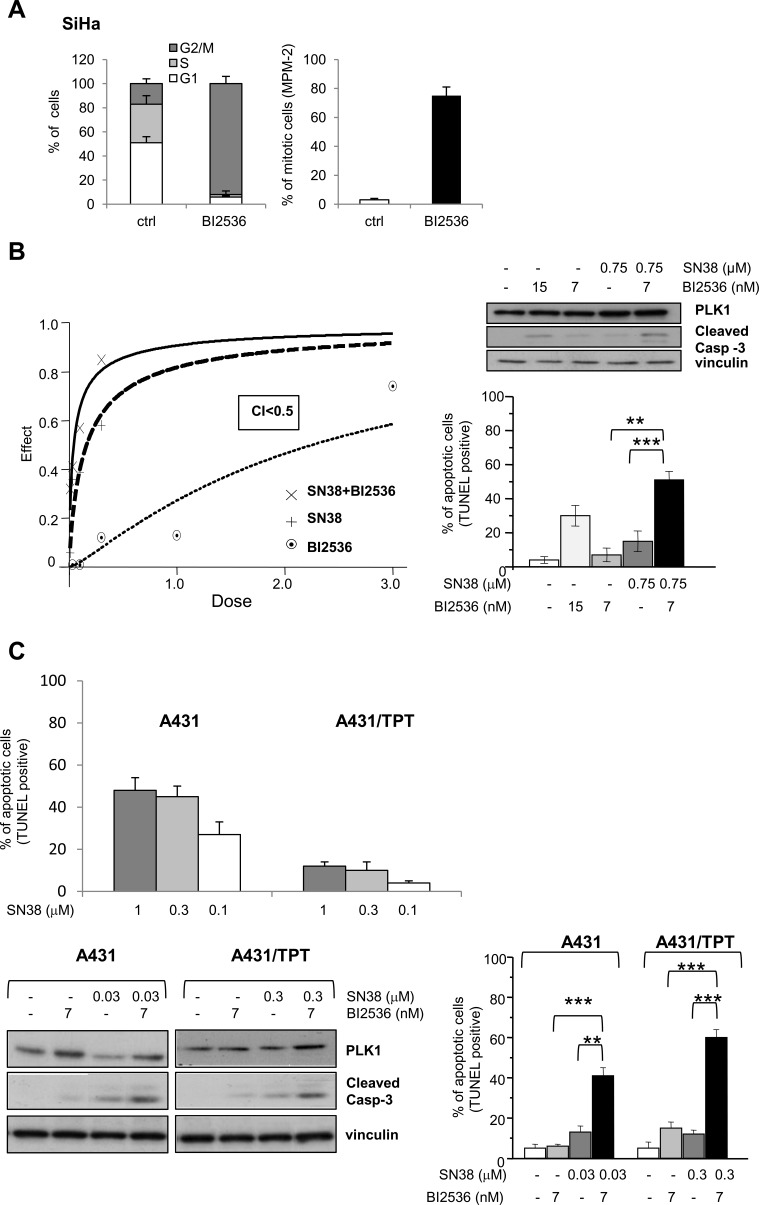
Synergistic antiproliferative effect and enhanced apoptotic response by combination treatment of SN38 with BI2536 in SCC cell lines A) The effect on cell cycle of the PLK1 kinase inhibitor BI2536 was first analyzed in SiHa cells exposed to the drug (15 μM) for 24h. Left, cell cycle phase distribution. Right, percentage of mitotic cells (MPM-2 immunofluorescence detection). B) SiHa cells were treated with solvent (−) or SN38 for 1 h and, 24h later, exposed to BI2536 for additional 48 h. SN38 and BI2536 were combined at a fixed molar ratio. Left panel, the antiproliferative effect was assessed by cell counting and the drug interaction evaluated by the combination index (CI) method, CI<1 indicates synergism. Dose-effect curves representative of one experiment out of three are shown. Right panels, apoptosis was assessed by TUNEL assay after treatment with BI2536 (IC_50_ and IC_80_) and SN38 (IC_50_) alone or in combination. In parallel with apoptosis detection, Western blot analysis was performed to reveal PLK1 levels and caspase-3 cleavage. C) A431 and A431/TPT cells were treated with solvent (−) or SN38 for 1h. In upper panel, quantification of TUNEL staining in the indicated SCC cells was performed 72 h after the end of treatment. Values are expressed as mean ± SD (n=3). In lower panels, the day after SN38 exposure, BI2536 was added where indicated. Left lower panel, after 24h, Western blot analysis was performed on whole-cell extracts to evidence levels of PLK1 and caspase-3 cleavage. Right lower panel, after 48 h from the addition of the PLK1 inhibitor, FACS analysis was performed to detect TUNEL-positive cells. Vinculin blot is shown as protein loading control. Columns and bars: mean values ± SD from three independent experiments. *P < 0.05; **P < 0.01, ***P < 0.001 by Student's t test.

Since the above experiments, based on molecular approaches, suggested PLK1 as an attractive target for sensitizing cells to CPTs, we investigated whether the responsiveness of SN38-resistant cellular models could be modulated by pharmacological inhibition of PLK1 enzymatic activity. BI2536, a highly selective PLK1 inhibitor [19, 34, 35] displayed comparable antiproliferative effects on both CPT-resistant and -sensitive cell lines (Suppl. Table 1). Similarly to the behavior observed in PLK1-silenced SiHa cells, PLK1 inhibition by BI2536 resulted in increased accumulation of cells with G2/M DNA content and mitosis (Fig. 4A).

We designed combination experiments with SN38 and BI2536 according to the Chou-Talalay method [36]. Whereas the simultaneous treatment of SiHa cells with the two drugs did not result in a favorable drug interaction, cell exposure to the CPT, followed 24h later by the PLK1 inhibitor, produced a synergistic inhibition of cell growth as evidenced by dose-effect plot and confirmed by combination index (CI) less than 1 (Fig. 4B). In addition, the combined treatment enhanced the apoptotic cell response with a significant increase of caspase-3 cleavage and TUNEL positivity (Fig. 4B). A similar effect was observed when the CPT-resistant rhabdomyosarcoma cells RD were exposed to the sequential combination treatment (Suppl. Fig 2A).

Next, we exploited the availability of a human SCC experimental model of acquired drug resistance consisting of the pair of isogenic cell lines A431 and the TPT-resistant variant (A431/TPT) cross-resistant to SN38 *in vitro* ([24] and Fig. 4C) and to CPT11 *in vivo* [37]. Again, in this system, the sequential exposure to SN38 and BI2536 resulted in a synergistic interaction (Suppl. Fig. 2A). Moreover, a significant apoptosis increase was observed in both sensitive and resistant cells after treatment with equitoxic concentrations of the CPT (Fig. 4C).

These findings indicated that inhibition of PLK1 enzymatic activity could enhance apoptosis in tumor cell lines characterized by intrinsic or acquired resistance to CPTs.

### CPT11 and BI2536 cooperate in potentiating the antitumor effect against SCC xenografts

**Figure 5:**
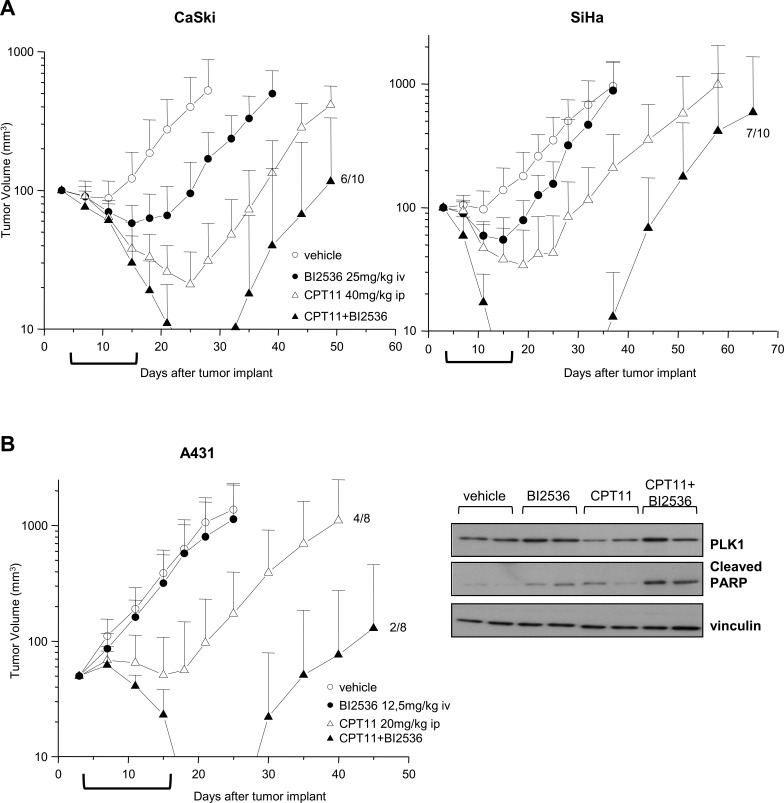
Enhancement of antitumor efficacy against SCC xenografts by combined treatment of CPT11 with BI2536 A) Mice bearing Caski or SiHa s.c. tumors were administered ip with 40 mg/kg CPT11 with an intermittent schedule (q4dx4). BI2536 was injected iv at 25 mg/kg 24h after CPT11 with the same intermittent schedule (q4dx4). Control mice received the drug's vehicles. B) Left, mice bearing A431 tumors were administered with 20 mg/kg CPT11 ip and 12.5 mg/kg BI2536 iv with intermittent sequential schedule as in A). Tumor volumes were measured twice a week and reported as means ± SD. Brackets under abscissas indicate the treatments’ timeframe. Right, A431 tumors from two mice/groups treated with single dose of vehicle, drugs alone or in combination, were removed after 24h from the last treatment. Tumors were lysed and analyzed by Western blotting for PLK1 and cleaved PARP levels. Vinculin levels show equivalent loading of tumor lysates. In groups of animals exhibiting stable tumor regression, the fraction of regrowing tumors is indicated.

The antitumor efficacy of CPT11 and BI2536 cotreatment was assessed in nude mice bearing SCC xenografts in a sequential schedule resembling the *in vitro* treatments (i.e. CPT11 injected ip on days 4; 8; 12; 16 followed, 24h after each CPT dose, by BI2536 iv). Administration of 40 mg/kg CPT11 alone to mice bearing CaSki xenografts resulted in a marked inhibition of tumor growth (94 % TVI), 1/10 complete response and maintenance of tumor growth delay for about one week after treatment interruption (Table 1 and Fig. 5A). As expected, SiHa tumors were less responsive to CPT11 treatment (84% TVI) with respect to CaSki tumors (Table 1 and Fig. 5B). In both SCC models, BI2536 administered as single agent at the 25 mg/kg dose, produced a moderate but significant inhibition of tumor growth (69% TVI, P<0.01, CaSki; 52% TVI, P<0.05, SiHa). In mice bearing CaSki tumors, the combination of CPT11 and BI2536 resulted in a significant implementation of TVI. Moreover, 8/10 mice experienced complete responses and 4/10 animals showed no evidence of disease at the end of the experiment 84 days after the last BI2536 delivery, testifying the curative potential of the combined treatment. Similarly, in SiHa tumor carrying mice, the combination yielded an impressive rate of tumor regressions with 10/10 mice experiencing complete responses and 3/10 animals without evidence of disease at the end of the experiment. In both SCC models, the increased efficacy of the combination versus single agent treatment was highlighted by the increased LCK, indicating the delayed recovery of the fraction of regrowing tumors.

**Table 1:** Antitumor activity of CPT11 and BI2536, alone or in combination, in nude mice bearing s.c. human squamous cell carcinomas

Model	Drug	Dose (mg/kg)	TVI%^1^	CR^2^	NED^3^	LCK^4^
CaSki	CPT11	40	94** (28)	1/10 ^ff^	-	1.2 (500)
	BI2536	25	69**	0/10 ^ff^	-	0.6
	CPT11 →BI2536	40 → 25	99	8/10	4/10	2
						
SiHa	CPT11	40	84* (22)	0/10 ^ff^	-	0.9 (500)
	BI2536	25	52**	0/9 ^ff^	-	0.2
	CPT11 → BI2536	40 → 25	100	10/10	3/10	1.4
						
A431	CPT11	20	87 (25)	4/8	4/8	1.1 (300)
	BI2536	12.5	18*	0/8 ^ff^	-	< 0.1
	CPT11 → BI2536	20 → 12.5	99	7/8	6/8	2.7
						
A431/TPT	CPT11	20	59** (22)	0/8	-	0.3 (300)
	BI2536	12.5	29**	0/8	-	0.1
	CPT11 → BI2536	20 → 12.5	83	0/8	-	0.8
						
	CPT11	40	96 (35)	3/8^f^	3/8	1.6 (300)
	BI2536	25	45**	0/8^ff^	-	0.4
	CPT11 → BI2536	40 → 25	100	8/8	5/8	1.8

1 Tumor volume inhibition % in treated over control mice. In parentheses, the day on which it was assessed.

2 Complete responses, i.e. disappearance of tumors lasting at least ten days.

3 No evidence of disease at the end of experiment, 100 days after tumor implant.

4 Gross log_10_ cell kill to reach the tumor volume reported in parentheses (mm^3^).

* P<0.05

** P< 0.01 by Student's t test

f P<0.05

ff P< 0.01 by Fisher's exact test, vs combination-treated mice.

Next, we tested the antitumor efficacy of the CPT11-BI2536 combined treatment against the couple of A431 and A431/TPT tumor xenografts. Given the known hypersensitivity of A431 to CPTs [24, 25, 37], CPT11 was delivered at the 20 mg/kg dose which produced in this model 87% TVI with 4/8 cured mice at the end of experiment (Table 1 and Fig. 5B). The combination of CPT with an almost ineffective dose of BI2536 resulted in a marked improvement of the antitumor activity with 99% TVI, 7/8 complete responses and 6/8 cured mice. Western blot analysis of A431 tumor specimens following a single administration of CPT11, followed by BI2536, revealed an increased PARP cleavage, indicating that the combination treatment was able to promote apoptosis also *in vivo* (Fig. 5B).

Administration of the same drug doses employed for the A431 model significantly improved the antitumor effect of the combination as compared to single agent treatments, also in mice bearing A431/TPT tumors, although without recording complete responses (Table 1). When higher doses of both drugs were used, an even improved benefit was observed with 100% of complete responses and 5/8 cures in mice receiving both drugs. Of note, the CPT11-BI2536 cotreatment was well tolerated (body weight loss <10% and no lethal toxicity). Thus, the addition of the PLK1 inhibitor to CPT11 provided a remarkable improvement of the therapeutic efficacy in all the SCC models tested.

## DISCUSSION

In this study, we provided preclinical rationale and mechanistic insights into a drug combinatory approach based on the use of PLK1 inhibitors to improve CPT-based antitumor therapies.

In previous studies designed to investigate the cell response to a novel Top1 poison, ST1968, we noticed that the susceptibility of human SCC and ovarian cancer cells to an early and significant CPT-induced apoptosis was associated with a marked reduction of the PLK1 protein [23]. Here, we assessed the concomitance of an efficient CPT-induced cell death and PLK1 downmodulation in a panel of SCC and pediatric sarcoma cell lines, and confirmed that PLK1 levels were not modulated in cells resistant to CPT-induced apoptosis.

PLK1 is a serine/threonine kinase that finely controls mitosis by regulating the activity of the anaphase-promoting complex/cyclosome (APC/C) and, ultimately, cell division [8, 12, 16]. In a wide range of pediatric tumors, including ESFTs characterized by high levels of PLK1, this kinase has been described as one of the most important survival kinases and a promising therapeutic target [26, 27]. By applying gene silencing and forced exogenous expression, we demonstrated that PLK1 acts as a prosurvival/antiapoptotic kinase also in SCC cells. These findings suggested that, even in this context, the mitotic kinase might represent a valuable target *per se*, and an exploitable target to foster chemotherapy-induced apoptosis. Indeed, the CPT11 active metabolite SN38 displayed an increased antiproliferative and proapoptotic activity in PLK1-silenced SiHa cells as compared to the intrinsically CPT resistant parental cells, thereby establishing a direct role for PLK1 in determining the cellular outcome in response to SN38. PLK1 is known to enhance cell tolerance to stress [16, 38]. Therefore, in conditions of stalled replication forks, known to be induced by CPTs [2, 3, 5], PLK1 inhibition is expected to induce stress sensitization by blocking the recovery from cell cycle arrest [38]. The failure of cells to downregulate PLK1 in response to CPTs can be related to a defective DNA damage checkpoint whereas it is not directly linked to overall level of protein expression (Suppl. Fig 2C). In fact, activation of a competent G2/M checkpoint requires a block of the pro-mitotic signals, including Cdc25A and PLK1 activity which is essential for the G2/M transition in cells attempting to recover from DNA damage [9, 16, 32]. Abrogation of PLK1 activity may occur by different strategies, including transcriptional repression and proteasome–mediated degradation [11, 12, 29, 30]. In our SCC cell lines, we did not find a direct correlation between inhibition of PLK1 transcription and PLK1 downregulation after SN38 treatment. In fact, a reduction of PLK1 mRNA levels was observed in both drug sensitive and resistant cell lines. Although a contribution of transcriptional inhibition to SN38-induced PLK1 downmodulation, as previously reported in response to CPT [31], cannot be excluded, the lower levels of ubiquitin binding to PLK1, observed in SiHa with respect to CaSki cells, were consistent with a defective activation/engagement of the cullin-based E3 ubiquitin ligases and proteasomal degradative processes [32]. Indeed, it has been reported that cells expressing a mutant form of *PLK1* resistant to APC/C^Cdh1^-mediated ubiquitination display a greater tendency to escape DNA damage checkpoint and enter mitosis despite the presence of DNA damage foci [11]. In SiHa cells which are unable to downregulate PLK1 following SN38 treatment, the DNA content, along with markers of cell cycle phase and the long-lasting hyperphosphorylation of RPA-2, indicated checkpoint escape and mitotic slippage despite the persistence of DNA damage.

Given the perspective of fostering the susceptibility to undergo apoptosis of either CPT-sensitive or –resistant tumor cells, we explored the therapeutic potential of a combination treatment with CPTs and PLK1 kinase inhibitors. PLK1 stands out as a promising target for molecular intervention in oncology and several small molecules targeting PLK1 are currently under clinical investigation [6, 7, 15, 18, 22, 39]. The dihydropteridinone BI2536 was the first PLK1 inhibitor investigated in patients with solid tumors, whereas current clinical studies favor the structurally related BI6727 (Volasertib, now entering Phase III) endowed with an improved pharmacokinetic profile [18, 21, 22, 40]. Pharmacological inhibition of PLK1 by BI2536 treatment in SCC cells resulted in the typical “Polo phenotype” [7] characterized by perturbed mitoses and apoptotic nuclei. Such phenotype resembled that observed following PLK1 RNA interference in the CPT-resistant SiHa cells, indicating that the impairment of the mitotic kinase enzymatic activity was sufficient to promote cell death. Accordingly, the combination treatment with SN38 and BI2536 resulted in a synergistic inhibition of cell growth and a marked enhancement of the apoptotic response. Moreover, the combination was able to implement antiproliferative effect and cell death in both the CPT-sensitive A431 cells and in A431/TPT cells characterized by acquired resistance to TPT and cross-resistant to SN38 ([24] and data herein).

Importantly, a striking enhancement of antitumor activity was obtained by CPT11 and BI2536 administered in combination to SCC xenografts bearing mice in a well-tolerated sequential schedule. Analysis of tumors showed enhanced apoptosis in mice treated with the combination, which was reflected in a remarkable number of complete tumor regressions. The improvement of tumor response also in models characterized by intrinsic and acquired resistance to CPTs further supported the therapeutic potential of the combination treatment. The combination of CPT11 with PLK1 targeting agents was previously assessed in neuroblastoma and colon carcinoma xenografts, although the molecular mechanisms underlying the antitumor efficacy were not elucidated [41, 42]. Of note, we performed our study in a panel of cell lines defective for p53 function as a consequence of gene mutation or Human Papilloma Virus (HPV) infection (http://p53.fr), [43]. A complex interplay exists between PLK1 and p53 involving mutual negative regulation at several molecular levels [16, 44, 45, 46]. Since there is evidence of a contribution of PLK1 in cellular transformation induced by viral oncoproteins including those from HPVs [16, 47], the combinatory approach proposed in our study might be of particular clinical interest in SCC.

Overall our data demonstrating a direct role for PLK1 in cell response to CPT11 highlight a novel rationale-based strategy to increase the therapeutic efficacy of CPT-containing regimens and to promote chemosensitization of resistant tumors.

## MATERIALS AND METHODS

### Cell lines and culture conditions

We used four human SCC cell lines, CaSki and SiHa, from uterine cervix and A431, from skin, along with the TPT-resistant subline (A431/TPT) selected in our lab [24], two human Ewing's sarcoma family of tumor cell lines SK-N-MC (Askin's tumor) and TC71 (Ewing's sarcoma) and the human embryonal rhabdomyosarcoma cell line RD. All SCC, SK-N-MC and RD cell lines were cultured in RPMI-1640 medium (Lonza, Verviers, Belgium), whereas the TC71 cell line was maintained in Iscove's modified Dulbecco's Medium (Lonza). All cell lines were grown in medium supplemented with 10% FBS and maintained in a humidified incubator with 5% CO_2_ at 37°C. The A431, CaSki and SiHa cell lines were obtained from American Type Culture Collection (Manassas, VA). The SK-N-MC cell line was kindly provided by R. Maggi (University of Milan, Italy), the RD cell line by A. Rosolen (University of Padua, Italy), and the TC71 cell line by M.C. Manara (Rizzoli Institute, Bologna, Italy). Cell lines were authenticated by single tandem repeat analysis by the AmpFISTR Identifiler PCR amplification kit (Applied Biosystems, Foster City, CA).

### Drugs and cell treatments

For *in vitro* studies, SN38 (Sigma Chemicals Company, St. Louis, MO) was dissolved in dimethyl sulfoxide (DMSO) and then diluted in sterile saline before use. The PLK1 inhibitor BI2536 (Axon Medchem B.V., Groningen, The Netherlands), was dissolved and diluted in DMSO (0.5% final concentration in culture medium). Exponentially growing cells were treated, the day after seeding, with increasing drug concentrations. Sarcoma cells were incubated in the continuous presence of SN38. SCC cells were exposed to the CPT for 1h then cells were washed and incubated in drug-free medium. All cell lines were exposed to BI2536 continuously.

The antiproliferative activity was evaluated after 72h from drug exposure by cell counting using a Coulter Counter (Coulter Electronics, Luton, UK). Drug concentrations able to inhibit cell proliferation by 50% (IC_50_) and 80% (IC_80_) were calculated from dose-response curves. The SN38/BI2536 combination treatment was assayed designing schedule treatments according to the Chou-Talalay method [36]. Cells were exposed to drugs at a constant-ratio in a sequential schedule consisting of exposure to SN38 for 1h followed, the day after, by the PLK1 inhibitor for additional 48 h. Drug interaction was analyzed by the CalcuSyn Software (Biosoft, Cambridge, UK) and expressed as combination index (CI). By this method, CI=1 indicates an additive effect, CI<1 synergism, and CI>1 antagonism.

### Western blot analysis and immunoprecipitation

Cells were processed for total protein extraction or immunoprecipitation as previously described in details [48]. Briefly, for immunoprecipitation of Plk1, cell lysates were incubated with protein A/G PLUS-Agarose (Santa Cruz Biotechnology, Santa Cruz, CA) and anti-Plk1 antibody (diluted 1:100, Cell Signaling Technology, Danvers, MA), for 24 h at 4 °C. Immunoprecipitates were then washed and eluted as described [48]. Lysates from frozen tumors were prepared as reported [37]. Briefly, tumors were pulverized by the Mikro-Dismembrator II (B. Brown Biotech International, Melsungen, Germany) and suspended in lysis buffer supplemented with protease and phosphatase inhibitors. Proteins from whole tumors, cell lysates or immunoprecipitates were separated by SDS-PAGE, transferred on nitrocellulose and analyzed for detection of specific proteins or phosphorylation as described [48]. Where indicates, band intensities were quantified by ImageJ software by pixel-integrated intensity.

### Antibodies

The antibodies used in the study were: anti-PLK1, anti-cleaved caspase-3, anti-cleaved PARP-1, anti-phospho-Histone H3 (Ser10) (Cell Signaling Technology); anti-vinculin (Sigma); anti-Cdc25A, anti-cyclin-B1 and -cyclin-D (Santa Cruz Biotechnology); anti-vinculin, anti-actin and anti-tubulin (Sigma); anti-RPA-2 (Neomarker, Union City, CS, USA), anti-γH2AX (Upstate Biotechnology, Lake Placid, NY, USA), anti-ubiquitin (Abcam, Cambridge, United Kingdom).

### Quantitative reverse-transcription PCR

Quantitative real-time PCR (qRT-PCR) was performed by the TaqMan PCR Kit (Applied Biosystems, Foster City, CA) according to manufacturer's instructions using TaqMan probes PLK1, Hs00153444_m1; GAPDH, Hs02758991_g1. The levels of templates in samples were determined through relative quantitation (RQ) using comparative CT (ΔΔCT) assay configuration. The data were normalized by the GAPDH housekeeping gene detection. Data analysis was performed with Sequence Detection Systems 2.2.2 software (Applied Biosystems).

### PLK1 loss and gain of function studies

Knock-down of PLK1 was performed using siRNAs for human PLK1 (ON-TARGET plus SMART pool) and as control, non-targeting siRNAs (On-TARGET plus non-Targeting Pool)(Dharmacon, Colorado, USA). CaSki and SiHa cells were transiently transfected with control or PLK1 siRNA at a final concentration of 100 nM using RNAimax (Invitrogen, Carlsbad, CA, USA). To increase *PLK1* expression levels, CaSki cells were transfected using a plasmid containing full length *PLK1* cDNA (Origene, Tema Ricerca, Bologna, Italy) in Lipofectamine 2000 (Invitrogen). Twenty four hours after the transfection with siRNA or plasmid, cells were treated with SN38.

### Mitosis and apoptosis detection

For quantification of mitoses, adherent and floating cells were collected, fixed in 4% paraformaldehyde and permeabilized with cold 100% methanol. Cells, spotted onto polylisinated microscope slides, were incubated overnight at 4°C with MPM-2 antibody recognizing mitosis-specific phosphoepitopes (1:200) (Upstate Biotechnology) followed by a goat anti-mouse IgG/rhodamine conjugated antibody (Upstate Biotechnology) diluted 1:1000. Slides, mounted with Mowiol (Sigma), were examined by a fluorescence microscope DMRB (Leitz Microscope, Wetzlar, Germany), and the number of cells positive for MPM-2 (mitotic) was counted (at least, 300 cells/sample).

Apoptosis was evaluated in floating and adherent cells by TUNEL (Terminal deoxynucleotidyl transferase dUTP Nick End Labeling) assay (Roche, Mannheim, Germany) according to manufacturer's instruction. Samples were analysed by flow cytometry.

### *In vivo* studies

All experiments were carried out using 8 weeks-old female athymic Swiss nude mice (Charles River, Calco, Italy). Mice were maintained in laminar flow rooms keeping temperature and constant humidity. Mice had free access to food and water. Experiments were approved by the Ethics Committee for Animal Experimentation of the Fondazione IRCCS Istituto Nazionale dei Tumori of Milan according to reported guidelines [49].

Tumor fragments obtained by serial sc passages were implanted on the right flank. Groups of 8-10 mice bearing one tumor sc were employed. Tumor growth was monitored by biweekly measurements of tumor diameters with a Vernier caliper. Tumor volume (TV) was calculated according to the formula: TV (mm^3^) = d^2^xD/2 where d and D are the shortest and the longest diameter, respectively.

CPT11, dissolved in distilled water, was delivered ip. BI2536, dissolved in HCl and diluted in saline (0.01N HCl final concentration), was administered iv. The two compounds were delivered in a volume of 10 ml/kg of body weight every 4 days for 4 times (q4dx4) giving BI2536, in combination experiments, 24h after CPT11. Treatments started three days after tumor implant, when nodules were just palpable. The efficacy of drug treatments was assessed as: tumor volume inhibition percentage (TVI %) in treated versus control mice, calculated according to the formula: TVI% = 100-(mean TV treated/mean TV control x 100); complete regressions (CR), i.e. disappearance of the tumors lasting at least ten days after the end of treatments; no evidence of disease (NED), i.e. mice without tumors at the end of the experiment (100 days after tumor implant); log_10_ cell kill (LCK) calculated employing the formula: LCK = (T-C)/(3.32xdT), where T and C are, respectively, the mean times (days) required by drug-treated and control tumors to reach the same volume. DT is the doubling time of control tumors. Drug tolerability was assessed as body weight loss percent during treatment, and lethal toxicity, i.e., any death in treated groups occurring before the end of experiment.

### Statistical analyses

Analyses by the Student's 2-tailed t test were performed using the GraphPad Prism software, version 4.0 (GraphPad Prism Inc., San Diego, CA). P values < 0.05 were considered significant. In antitumor activity studies, Student's t and Fisher's exact test (two-tailed) were used for statistical comparison of tumor volumes and complete responses to treatments, respectively, in mice.

## SUPPLEMENTAL MATERIAL, TABLES AND FIGURES


