# Supplementary material for: PLK1 is a critical determinant of tumor cell sensitivity to CPT11 and its inhibition enhances the drug antitumor efficacy in squamous cell carcinoma models sensitive and resistant to camptothecins
Source: Oncotarget. 2015 Mar 12;6(11):8736–49. doi: 10.18632/oncotarget.3538 (PMC4496180; doi:10.18632/oncotarget.3538)
Supplement: Supplementary file 1 [file oncotarget-06-8736-s001.pdf]

PLK1 is a critical determinant of tumor cell sensitivity to CPT11 and its inhibition enhances the drug antitumor efficacy in squamous cell carcinoma models sensitive and resistant to camptothecins

Supplemental Material

Supplemental Table 1: Antiproliferative activity of SN38 and BI2536 in SCC cell lines (IC_{50})

Cell line	SN38 (uM)	BI 2536 (nM)
CaSki	0.23 ± 0.11	4.3±0.4
SiHa	1.4 ± 0.6	4.8±2.1
A431	0.023 ± 0.017	5.4±0.2
A431/TPT	0.17 ± 0.04	7.1±0.1

Cell growth inhibition was assessed by cell counting 72h after 1h of treatment with SN38 or after 72 h of continuous exposure to BI2536. Mean IC_{50} values ± SD from at least three experiments performed in duplicate are reported.

Supplemental Table 2: Antiproliferative activity of SN38 in sarcoma cell lines

Cell line	IC_{50} (nM)	IC_{80} (nM)
TC-71	0.71±0.15	1.4±0.3
SK-N-MC	0.23±0.11	3.4±0.7
RD	37±9	38±3

Cell growth inhibition was assessed by cell counting after 72h of continuous exposure to the drug. Mean values ± SD from at least three experiments performed in duplicate are shown.

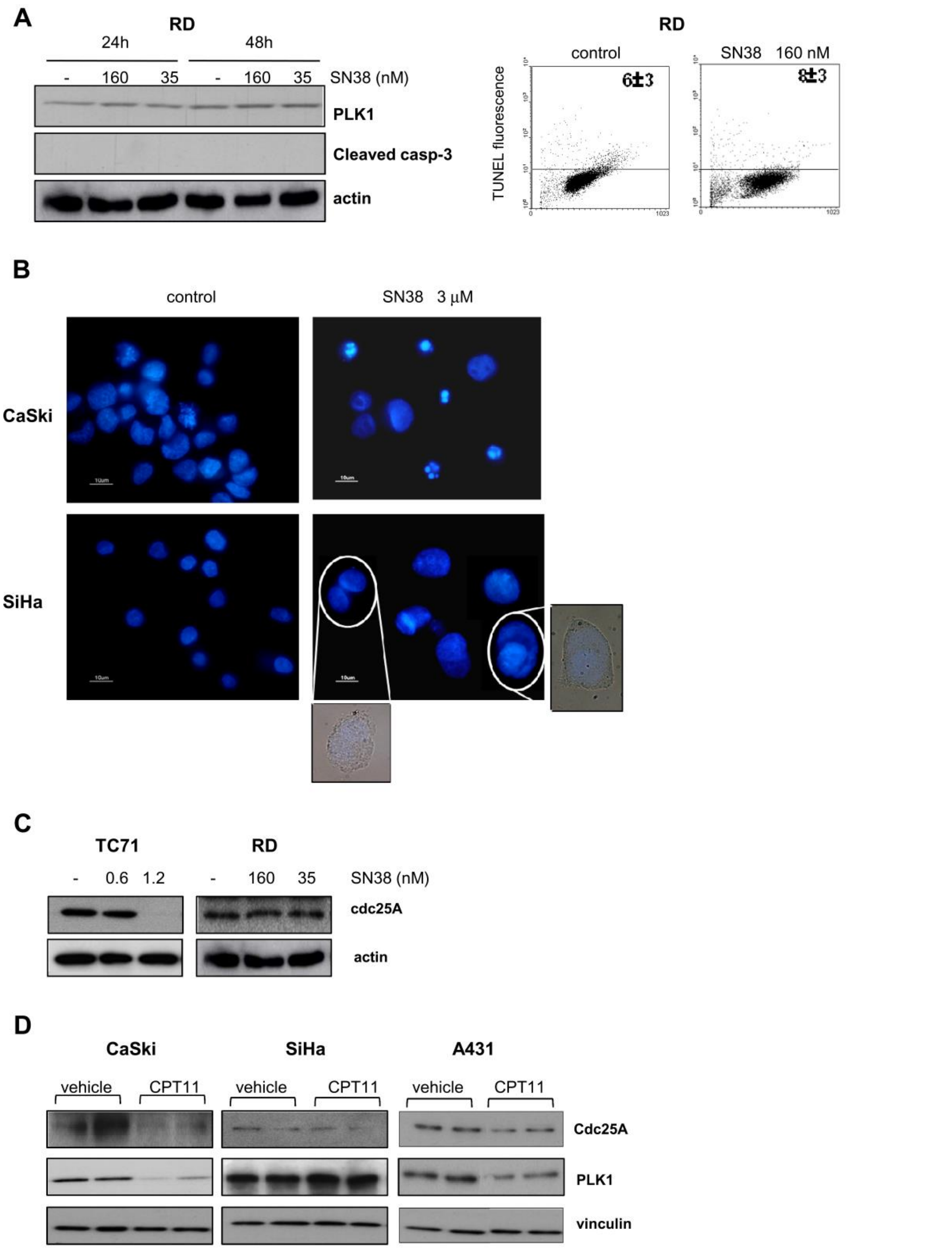

Suppl. Fig. 1

Supplemental Fig. 1: Effects of SN38 on rhabdomyosarcoma and SCC cell lines. A) The

rhabdomyosarcoma cells RD were incubated with different concentrations of SN38 (IC_{50} and IC_{80}) and processed after 24 and 48 h for Western blot analysis to assess the expression of PLK1 and cleaved caspase-3 (left panel) or, after 72 h, for TUNEL assay to assess apoptosis (right panel). Actin is shown as a control of protein loading. Dot plots from one experiment report mean percentages ± SD obtained in three independent experiments. B) Nuclei staining by Hoechst in CaSki and SiHa cells treated for 1 h with 3μM SN38. Cells were fixed in paraformaldehyde 24 h after the end of treatment. Insets show respective phase-contrast images of two representative polynucleated cells. C) Western blot analysis of Cdc25A expression in sarcoma cells exposed to SN38 for 24h. D) Cdc25A and PLK1 expression levels in SCC from mice treated with CPT11 (40 mg/kg i.p). Twenty-four hours later treatment, tumors were removed and protein extraction was performed as described in 'Materials and methods'. Antibodies to vinculin or actin were used to control for protein loading.

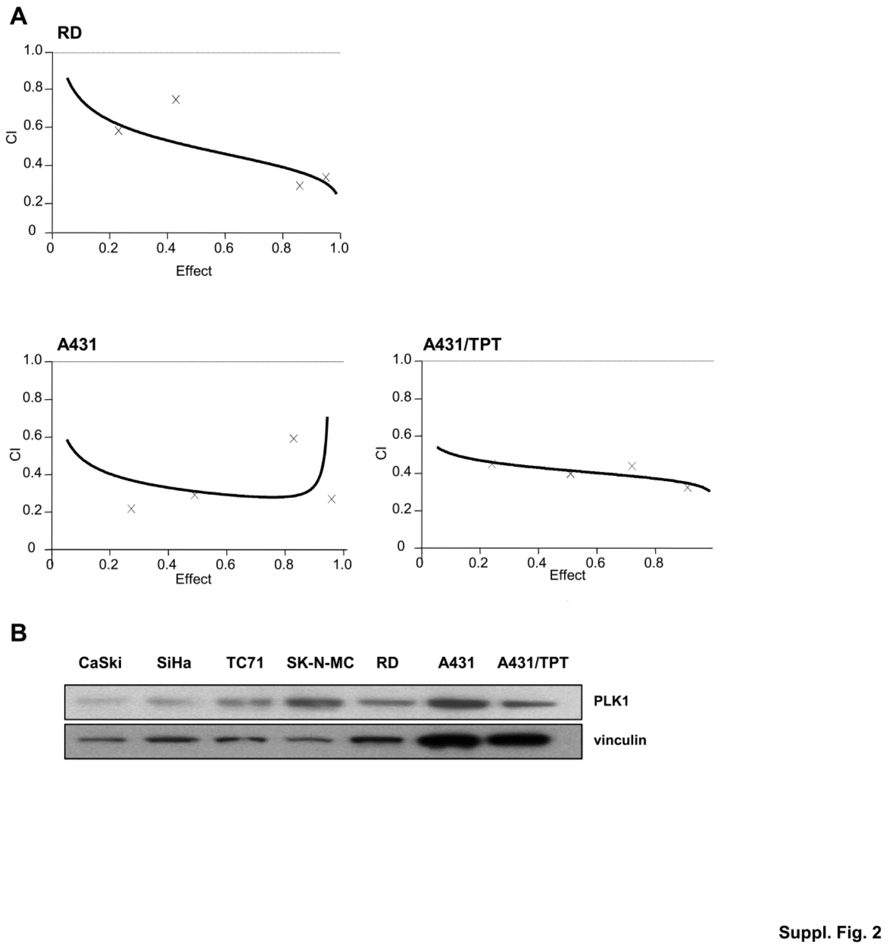

Supplemental Fig. 2: Synergistic antiproliferative effect by the combination of SN38 with BI2536 in CPT-sensitive and –resistant tumor cell lines. A) Synergistic antiproliferative effect of the combination of SN38 with BI2536 in the CPT-resistant rhabdomyosarcoma RD and SCC A431/TPT cell lines and in CPT-sensitive A431 cell line.Cells were treated with SN38 for 1 h and, 24 h later, exposed to BI2536 for additional 48 h. SN38 and BI2536 were combined in a range of concentrations at a fixed molar ratio. The antiproliferative effect was determined by cell counting and the drug interaction evaluated by the combination index (CI) method (CI<1 indicates synergism). B) PLK1 protein expression levels in the panel of SCC and sarcoma cell lines. Cell lysates were subjected to immunoblotting using the indicated antibodies. Vinculin was used as loading control.
